# Oral erythroplakia and oral erythroplakia-like oral squamous cell carcinoma – what’s the difference?

**DOI:** 10.1186/s12903-023-03619-2

**Published:** 2023-11-13

**Authors:** Jenny Öhman, Ayelet Zlotogorski-Hurvitz, Alex Dobriyan, Shoshana Reiter, Marilena Vered, Jaana Willberg, Carlo Lajolo, Maria Siponen

**Affiliations:** 1 Department of Oral Medicine and Pathology, Institute of Odontology, The Sahlgrenska Academy, University of Gothenburg, Box 450, Gothenburg, 40530 Sweden; 2 Department of Clinical Pathology and Cytology, Sahlgrenska University Hospital, Blå Stråket 5, Gothenburg, 41345 Sweden; 3 Department of Oral Pathology, Oral Medicine and Maxillofacial Imaging, School of Dentistry, Tel Aviv University, P.O. Box 39040, Tel Aviv, 6997801 Israel; 4 Department of Oral and Maxillofacial Surgery, Rabin Medical Center, Jabotinski St 39, Petah Tikva, 49100 Israel; 5 Department of Oral and Maxillofacial Surgery, The Chaim Sheba Medical Center, Derech Sheba 2, Tel Hashomer, Ramat Gan, 52621 Israel; 6 Institute of Pathology, The Chaim Sheba Medical Center, Derech Sheba 2, Tel Hashomer, Ramat Gan, 52621 Israel; 7 Department of Oral Pathology and Oral Radiology, Institute of Dentistry, Faculty of Medicine, University of Turku, University of Turku, Turku, 20014 Finland; 8 Department of Pathology, Turku University Hospital, P.O. Box 52, Turku, 20521 Finland; 9 Head and Neck Department, School of Dentistry, “Fondazione Policlinico Universitario A. Gemelli - IRCCS”, Università Cattolica del Sacro Cuore, Largo A. Gemelli, 8, Rome, 00168 Italy; 10 Institute of Dentistry, University of Eastern Finland, Kuopio Campus, P.O. Box 1627, Kuopio, 70211 Finland; 11 Department of Oral and Maxillofacial Diseases and Oral Health Teaching Clinic, Kuopio University Hospital, P.O. Box 1711, Kuopio, 70211 Finland; 12 Cancer and Translational Medicine Research Unit, University of Oulu, P.O. Box 8000, Oulu, 90014 Finland

**Keywords:** Erytroplakia, Oral erythroplakia, Oral squamous cell carcinoma, Oral potentially malignant disorder

## Abstract

**Background:**

Oral erythroplakia (OE) is a rare oral potentially malignant disorder, that has a high rate of malignant transformation. The definition of OE still lacks uniformity. In particular, lesions that look clinically like erythroplakias, but are histopathologically diagnosed as squamous cell carcinomas are still sometimes called erythroplakias. The purpose of this study is to present demographic and clinicopathologic features of a series of OEs and clinically oral erythroplakia -like squamous cell carcinomas (OELSCC), to study their differences and to discuss the definition of OE.

**Methods:**

A multicenter retrospective case series of OEs and OELSCCs. Descriptive statistics were used to analyze the data.

**Results:**

11 cases of OEs and 9 cases of OELSCCs were identified. The mean age of the OE patients was 71 years and 72.7% were female, while the mean age of the OELSCC patients was 69 years, and all were female. 9% of the OE and 22% of the OELSCC patients had smoked or were current smokers. 72.7% of the OEs and 55.5% of OELSCCs were uniformly red lesions. 63.6% of the OE and 22% of the OELSCC patients had a previous diagnosis of oral lichenoid disease (OLD). The malignant transformation rate of OE was 9% in a mean of 73 months.

**Conclusions:**

OE and OELSCC may arise de novo or in association with OLD. Tobacco and alcohol use were not prevalent in the present cases. The clinical features of OEs and OELSCC are similar, but symptoms, uneven surface and ulceration may be more common in OELSCCs than in OEs. Clinical recognition of OE is important since it may mimic other, more innocuous red lesions of the oral mucosa. The diagnosis of OE requires biopsy and preferably an excision. Clarification of the definition of OE would aid in clinical diagnostics.

**Supplementary Information:**

The online version contains supplementary material available at 10.1186/s12903-023-03619-2.

## Background

Oral erythroplakia (OE) is a rare lesion of the oral mucosa belonging to the oral potentially malignant disorders (OPMD) [1] and having a malignant transformation rate (MTR) of 19.9–45% [2–4]. The prevalence of OE is estimated to be 0.17% [5]. Clinically, OE presents as an often sharply-demarcated, solitary red patch on the oral mucosa that may be situated at a slightly lower level than the surrounding mucosa [6, 7]. The colour of the lesion is typically bright (fiery) red, and the surface has a matte smooth, velvety or granular appearance [1, 7, 8]. The soft palate, floor of the mouth and buccal mucosa are the most common locations of OE [7]. The etiologic factors of OE are thought be similar to the more common OPMD, oral leukoplakia (OL), and include tobacco, betel quid (areca nut) and alcohol use [9, 10]. It is said in the literature that around 90% of the uniformly red erythroplakias have oral dysplasia, carcinoma in situ or invasive carcinoma on first biopsy [11] and that most OEs show either high-grade dysplasia or squamous cell carcinoma (SCC) at the time of diagnosis [1]. However, a widely used definition of OE is “a red patch that cannot be clinically or pathologically diagnosed as any other definable disease” (Supplementary table). This suggests that a biopsy is always necessary to diagnose erythroplakia. If a clinically erythroplakia-like lesion has invasive carcinoma histopathologically, it cannot be called erythroplakia by definition.

The term erythroplakia derives from the term ‘erythroplasie’, probably first used by the French dermatologist Queyrat to describe a bright red, velvety, sharply defined precancerous lesion of the glans penis [12]. He coined the term by analogy to the French term ‘leucoplasie’. As suggested by Shear [13], the English language version of ‘erythroplasie’ would be erythroplakia (analogously to leukoplakia). First plausible description of oral mucosal erythroplakias (erytroplasia) were published in 1963 by Shedd et al. [14]. In 1948, Sachs and Sachs reported on 10 cases of erythroplasia of Queyrat of the glans penis and mentioned seeing erythroplasia also on the buccal mucosa. However, they saw no microscopic or clinical evidence for precancerous or malignant change in any of their cases, and the diagnosis of OE could therefore be questioned [15]. Erythroplastic appearance in an oral mucosal lesion or erythroplasia (rather than leukoplakia) has been reported also as a possible manifestation of early, asymptomatic oral SCC [16, 17].

It is recognized that the definition of OL and OE remains unsatisfactory [7, 18, 19]. In the context of oral leukoplakias/erythroplakias, the mixed red and white lesions are generally classified as erythroleukoplakias [19–21] (Supplementary table). However, some experts describe erythroplakia as a *predominantly* red lesion of the oral mucosa that cannot be characterized clinically or pathologically as any other definable lesion [1, 22, 23]. In fact, the 2017 WHO Classification of Head and Neck Tumours defines OE in relation to leukoplakia: “ ’Leukoplakia’ is a clinical term used to describe white plaques of questionable risk, once other specific conditions and other oral potentially malignant disorders (OPMD) have been ruled out, which normally requires biopsy. Leukoplakias can be homogeneously white or predominantly white with nodular, verrucous or red areas. Predominantly white examples with red areas are called erythroleukoplakias (speckled leukoplakias). Oral erythroplakia is defined equivalently, but as a red patch” [24].

Possibly due to its rarity and due to the historical practice of considering OE as the red counterpart of OL, it is defined in relation to OL, and often reported in studies in conjunction with OL. Extracting data of OEs from these studies is often impossible. Reports and studies focusing solely on OE are rare. In addition, erytroplakia-like lesions with invasive carcinoma occasionally have been reported as OEs. The purpose of this case series is therefore to present the demographic, clinical and histopathologic features of OEs and to compare the relevant features to clinically oral erythroplakia-like squamous cell carcinomas (OELSCC) in a predominantly European population. In addition, the aim of this report is to discuss the definition of OE.

## Methods

A retrospective search for cases with the diagnosis of oral erythroplakia was done in the participating centers. The diagnoses of OE and OELSCC were done by taking into account the clinical and histopathologic features of the cases. The authors agreed on the diagnosis of all the cases. Data on patient demographic characteristics, smoking and alcohol use history, oral mucosal disease history, OE and OELSCC clinical and histopathological features, treatment, follow-up and lesion recurrence, and OE malignant transformation was collected. Descriptive statistical methods were used to analyze the data.

The study was carried out according to the guidelines of the Declaration of Helsinki. Approval for the study was granted and the need for consent was waived by the ethical committees of the Northern Ostrobothnia Hospital District, Finland (46/2013), the Regional Ethical Review Board in Gothenburg, Sweden (729 − 18), Sheba Medical Center, Israel (6666-19-SMC) and Tel Aviv University, Israel (no official number). Kuopio University Hospital granted organization permit (238/2016) for the study. A written informed consent was obtained from the study participants at Università Cattolica del Sacro Cuore, Italy and Turku University Central Hospital, Finland.

## Results

Eleven cases of OEs and nine cases of OELSCCs were found. The mean age of the OE patients was 71 years and 73% were female (Tables 1 and 2). The mean age of the OELSCC patients was 69 years and 100% were female (Tables 1 and 3). None of the OE patients were smokers; one reported having smoked in the past and two (18%) stated that they had never smoked. 78% of the OELSCC patients were non-smokers, and 5 patients (55.5%) stated that they had never smoked. None of the patients reported using smokeless tobacco products. 62.5% (5/8) of the OE patients and 37.5% (3/8) of the OELSCC patients reported using alcohol. Some notion about the amount of alcohol used could be found for 5 OE patients (“very little”, “little”, “several times a week”, “4 cl per week” and “21 cl per week”) and for 2 OELSCC patients (“2–3 portions per week” and “occasionally”).

**Table 1:** Summary of the main demographic, clinical and histopathologic characteristics of the oral erytroplakia (OE) and the clinically oral erythroplakia -like oral squamous cell carcinoma (OELSCC) cases

	OE	OELSCC
Age mean (range)	71 (54-91)	69 (48-82)
Gender n (%)		
Female	8 (72.7)	9 (100)
Male	3 (27.3)	0 (0)
Smoking n (%)		
Yes	0 (0)	2 (22.2)
No	11 (100)	7 (77.8)
Lesion location^a^ n (%)		
Buccal mucosa	5 (45.4)	2 (22.2)
Gingiva	3 (27.3)	3 (33.3)
Tongue	2 (18.2)	2 (22.2)
Hard palate	1 (9.1)	2 (22.2)
Floor of mouth	0 (0)	1 (11.1)
Symptoms n (%)		
Yes	3 (27.3)	6 (85.7)
No	8 (72.7)	1 (14.3)
Histopathologic diagnosis of OE n (%)		
Mild dysplasia	1 (9.1)	n/a
Moderate dysplasia	1 (9.1)	n/a
Severe dysplasia	4 (36.4)	n/a
Carcinoma in situ	3 (27.2)	n/a
Other^b^	2 (18.2)	n/a
Follow-up (average in months)	72.7	33.1
Malignant transformation n (%)	1 (9.1)	n/a

^a^One OELSCC patient had lesion extending to two locations: buccal mucosa and gingiva

^b^Lichenoid inflammation and epithelial atypia (n=1), lichenoid reaction and ulceration (n=1)

n/a = not applicable

**Table 2:** Demographic and clinicopathologic features of the oral erythroplakia cases

	Country	Sex/age	Smoking	Alcohol use	Site	Clinical features	Incision biopsy diagnosis/ Diagnosis after excision	Size in mm	Symptoms	Treatment	Follow-up in months	Recurrence of OE	Malignant transformation
1	Finland	F/56	No	N/av	BM	Well-defined	SD/SD	N/av	Tenderness, especially when eating spicy food and citruses	SE	180	No	No
2	Finland	F/81	No	Yes, “very little”	BM	Mostly well-defined	(1) Severe inflammation, epithelial atypia and ulceration, (2) Lichenoid inflammation, severe inflammation, (3) Inflammatory atypia x 3, active inflammation x 2, eosinophilia, severe inflammation/MoD (HPV, p16 negative	17 × 33	Tenderness, pain when eating and on palpation	SE	56	No	No
3	Finland	F/78	Never	No	BM	Well-defined, situated slightly below the adjacent mucosa	CIS/CIS	5 × 5	Intermittent pain	SE	63	No	No
4	Finland	F/62	Never	Yes	G	Well-defined, situated slightly below the adjacent mucosa	(1) Lichenoid inflammation (BM), IF negative (G), (2) Lichenoid inflammation, (3) Lichenoid inflammation and epithelial atypia x 3, (4) IF negative/n/a	5 × 40	No	LE	42	No	No
5	Italy	F/81	No	No	BM	Well-defined	Severe dysplasia/n/a	20 × 30	No	LE	36	Yes	No
6	Sweden	F/78	No	N/av	G	Well-defined	CIS/CIS	20 × 30	No	SE	96	Yes	No
7	Sweden	M/76	No	Yes	HP	Well-defined, minor white areas	SD/SD	30	No	SE	96	Yes	Yes
8	Sweden	M/72	No	Yes	BM	Well-defined, white areas around the periphery	MoD/SD	20 × 30	No	SE	121	Yes	No
9	Sweden	F/57	Previously	N/av	G	Partly well-defined	LR/LR + MiD	20	No	SE	84	Yes	No
10	Sweden	F/54	No	No	T	Well-defined, minor white areas around the periphery	LR/Ulceration + LR	20	No	SE	24	No	No
11	Sweden	M/91	No	Yes	T	Well-defined	CIS/CIS	10	No	SE	2	No	No

CIS = carcinoma in situ, LR = lichenoid reaction, MiD = mild dysplasia, MoD = moderate dysplasia, OE = oral erythroplakia, SD = severe dysplasia, BM = buccal mucosa, FOM = floor of mouth, G = gingiva, HP = hard palate, T = Tongue, SE = surgical excision, LE = laser evaporation

N/a = not applicable

N/av = information not available

**Table 3:** Demographic and clinicopathologic features of the clinically oral erythroplakia-like oral squamous cell carcinoma cases

	Country	Sex/age	Smoking	Alcohol use	Site	Clinical features	Incision biopsy diagnosis/ Diagnosis after excision	Size in mm	Symptoms	Treatment	Follow-up in months	Recurrence of SCC
1	Finland	F/58	No	N/av	T	Mostly well-defined, peripheral minor white areas	SCC (microinvasive)/SCC (microinvasive)	N/av	Smarting, burning sensation	SE	92	Yes
2	Finland	F/73	Never	Yes	BM, G	Well-defined, situated slightly below the adjacent mucosa, peripheral minor white areas	SCC (gingiva)/SCC (gingiva)	35	Smarting sensation, pain radiating to ear and maxillary sinus	SE	59	No
3	Finland	F/48	Yes	Occasionally	FOM	Mostly poorly defined, associated ulceration	SCC/SCC	15 × 25	N/av	SE	5	N/av
4	Israel	F/82	Never	No	BM	Well-defined, sinuous borders	(1) VH with dysplasia, (2) SD/SCC	40	No	SE	N/av	N/av
5	Israel	F/76	Never	No	T	Well-defined, peripheral minor white areas	(1) SD, (2) CIS/SCC	30	Pain	SE	24	No*
6	Israel	F/69	No	No	HP	Well-defined, minor ulceration around the periphery	SCC/SCC	15	N/av	SE	12	No
7	Italy	F/80	Never	No	HP	Well-defined, situated below the adjacent mucosa	SCC/SCC	20 × 35	Dental mobility	N/av	N/av	N/av
8	Italy	F/79	Never	No	G	Mostly well-defined, sinuous borders, easy bleeding, associated ulceration	SCC/SCC	20 × 35	Burning sensation, pain	SE	6	N/av
9	Sweden	F/56	Yes	Yes	G	Well-defined, situated below the adjacent mucosa, peripheral minor white areas	SCC/Reactive changes	20 × 10	Smarting sensation	SE	34	Neck metastasis after 20 months

CIS = carcinoma in situ, MiD = mild dysplasia, MoD = moderate dysplasia, SCC = squamous cell carcinoma, SD = severe dysplasia, VH = verrucous hyperplasia

BM = buccal mucosa, FOM = floor of mouth, G = gingiva, HP = hard palate, T = Tongue

SE = surgical excision

N/av = information not available

*Carcinoma in situ after 12 months

The most common locations for OE were buccal mucosa (45%) and gingiva (27%), followed by tongue (18%) and hard palate (9%) (Tables 1 and 2). The most common site for OELSCC was gingiva (33%), and buccal mucosa, tongue or hard palate were affected in 22% of the cases (1 case had both buccal mucosal and gingival involvement) (Tables 1 and 3). One case (11%) of OELSCC was located in the floor of the mouth.

**Fig. 1:**
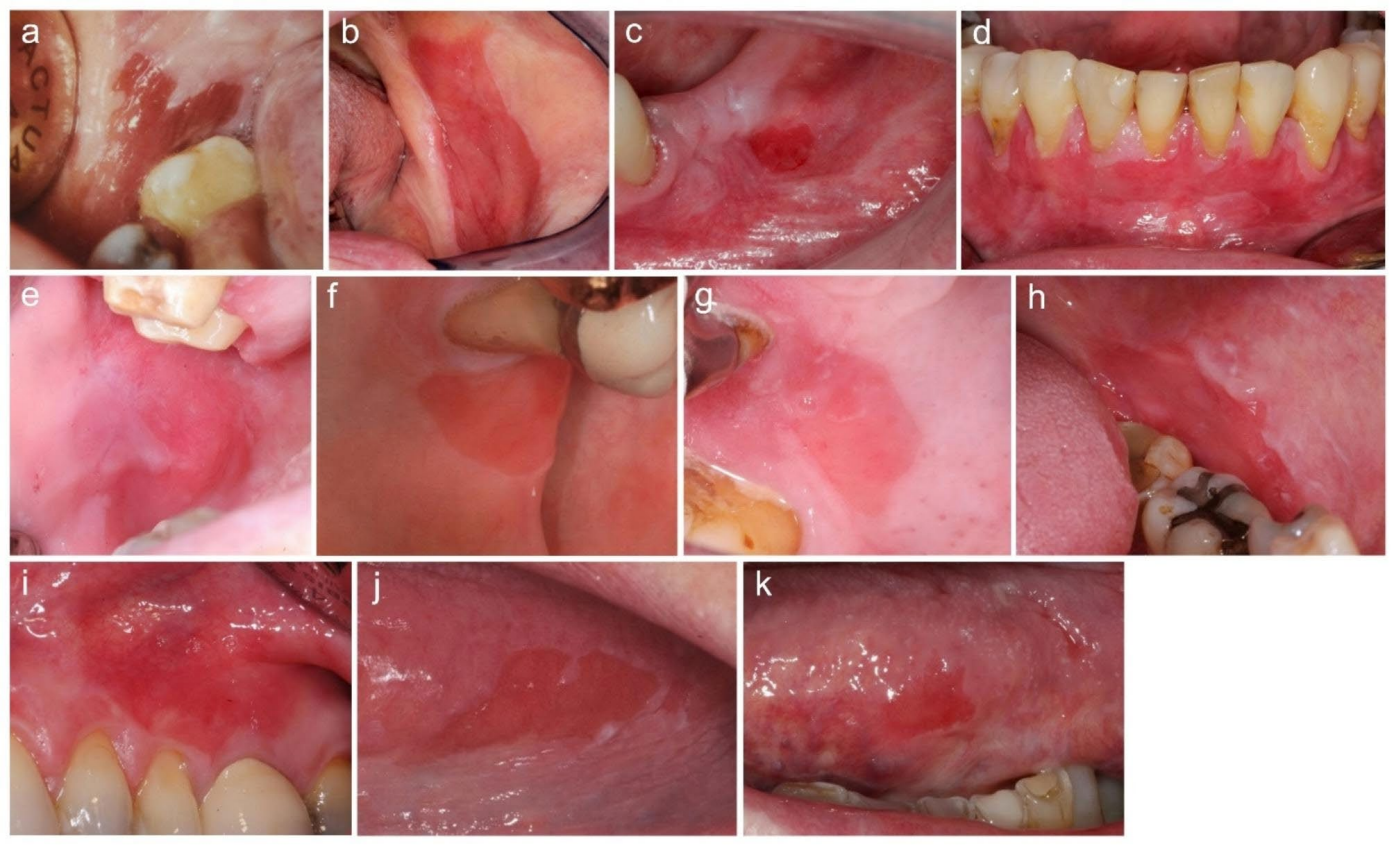
The clinical presentation of oral erythroplakias (OE) (**a-k**, patients 1–11). The most common location of OEs was buccal mucosa. Most OEs were well-defined, and all had a smooth surface

73% (n = 8) of the OEs were uniformly red lesions, and 27% (n = 3) had minor white areas associated with the lesion (Table 2; Fig. 1). 82% (n = 9) of the OEs were well-defined, and 18% (n = 2) were mostly or partly well-defined. Two of the lesions were described as being situated at a slightly lower level than the surrounding mucosa. The surface of the OEs was bright red, matte or shiny and smooth. The size of OEs ranged from 5 mm in diameter to 40 mm in greatest dimensions (Table 2).

**Fig. 2:**
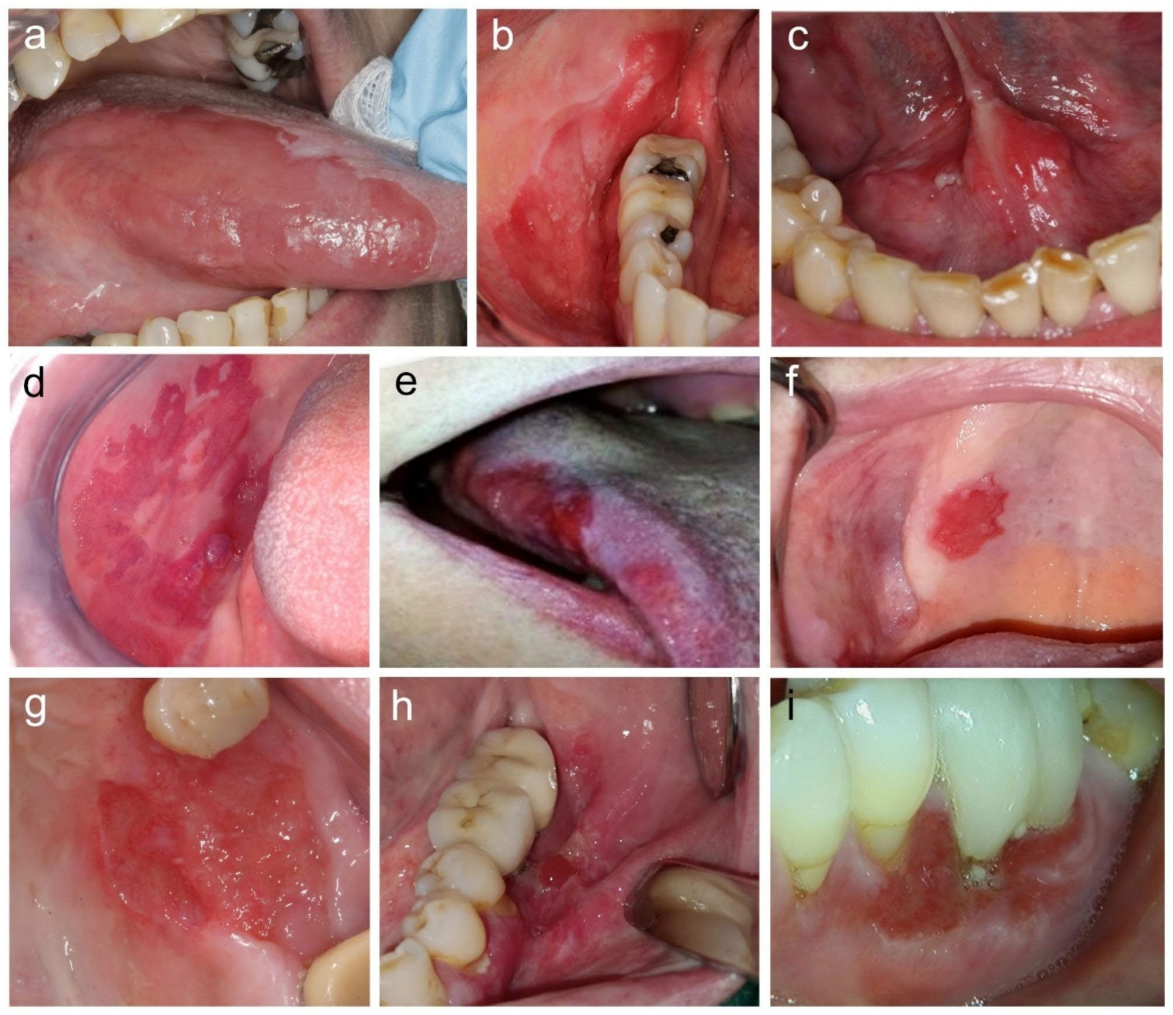
The clinical presentation of oral erythroplakia-like squamous cell carcinomas (OELSCC) (**a-i**, patients 1–9). The most common location of OELSCCs was gingiva. Most OELSCCs were well-defined. Some lesions had an uneven surface and/or associated ulceration

22% (n = 2) of the OELSCCs were uniformly red lesions, 44% (n = 4) had minor white areas and 33% (n = 3) had some ulceration associated with the lesions (Table 3; Fig. 2). 67% (n = 6) of the OELSCCs were well-defined, 22% (n = 2) were mostly well-defined and 11% (n = 1) were a mostly poorly-defined lesions. The surface of the OELSCCs was bright red, matte or shiny and some lesions had a somewhat uneven surface. One lesion was clearly depressed below the surface of the surrounding mucosa. The size of OELSCCs ranged from 15 mm in diameter to 40 mm in greatest dimensions (Table 3).

27% (n = 3) of the OE patients and 86% (6/7) of the OELSCC patients experienced symptoms associated with the lesions (Tables 2 and 3).

Seven (64%) of the OE patients had a previous diagnosis of oral lichenoid disease (OLD), either oral lichen planus (OLP) (n = 5) or oral lichenoid lesions (OLL) (n = 2). Of the OELSCC patients, 22% had been diagnosed previously with OLP (n = 1) or OLL (n = 1).

82% (n = 9) of the OEs were histopathologically dysplastic and two cases (18%) showed lichenoid inflammation (associated with either epithelial atypia or ulceration) (Table 2). 64% of the OEs were diagnosed as severe dysplasia (n = 4) or carcinoma in situ (n = 3), 9% (n = 1) as moderate dysplasia and 9% (n = 1) as mild dysplasia. Histopathologic diagnosis remained unchanged between the first incisional biopsy and the excisional biopsy in 67% (6/9) of the cases but changed to a more severe histopathologic diagnosis after excision biopsy in 33% (3/9) of the cases. Of the OELSCCs, two were diagnosed as severe dysplasia or carcinoma in situ in the first incisional biopsy but diagnosed as invasive SCC after excision (Table 3).

Nine of the OEs were treated with surgical excision, and two cases with laser evaporation. The mean follow-up of the OE patients was 73 months (range 2-180 months). Recurrence of OE was observed in 45% (n = 5) of the cases and malignant transformation to SCC (T3N0M0) occurred in one case after 96 months of follow-up. The mean follow-up of OELSCC patients was 33 months (range 5–92). Recurrence of SCC was found in 25% (1/4) of the OELSCC cases where the information was available. One OELSCC patient developed no local recurrence but a neck metastasis during follow-up.

## Discussion

The definition of oral erythroplakia has changed relatively little over the years, and the main differences in the definition have involved the clinical description of oral erythroplakia as either purely red (sometimes called homogenous erythroplakia) or as a predominantly red lesion (meaning it could contain minor areas of e.g. white appearance) (Supplementary table). Although almost all definitions of OE include the notion that it “cannot be characterized clinically or pathologically as any other definable disease”, many reports on OE include cases that were either not biopsied [9] or actually diagnosed histopathologically as squamous cell carcinomas [11, 25–27].

It would perhaps be better to define OE for example as a “red or a predominantly red lesion of the oral mucosa that cannot be diagnosed as any other lesion and that histopathologically features epithelial dysplasia in almost all cases”. That would emphasize its dangerous nature and the need to histopathologically examine the whole lesion.

In the present study, the vast majority of the OE patients were female, while it is reported that OE is found equally in females and males [9, 11]. The relatively large proportion of patients with previous OLD, that is more common in females, may partly explain the gender distribution in our case series. All the OELSCC patients were female, in contrast to the gender distribution of oral SCC in general. OE occurs mainly in the middle aged and older age groups [7], which was found also in the present study.

Although tobacco use and alcohol drinking are suspected to be predisposing factors for OE [7, 9] and oral SCC, none of the OE patients were current smokers (one had smoked in the past) and only 22% of the OELSCC patients were smokers. The small proportion of smokers in the present case series may be partly explained by the gender distribution, since in many countries, most of the smokers are males. Majority of the OE patients reported using small or very moderate amounts of alcohol, while less than 40% of the OELSCC patients used alcohol. None of the patients used smokeless tobacco products or areca nut/betel quid, which are reported to be risk factors for OE in Indian population [9]. Although the number of cases is small in the present study, the findings suggest that other factors than tobacco, areca nut and alcohol use are also contributing to the development of OE.

It is reported before that *erytroplakic* lesions may arise in association with OLL or OLP [6, 28]. Indeed, in the present case series, over 60% of the OE patients and over 20% of the OELSCC patients had a previously diagnosed OLD. It is therefore possible that OLD predisposes to OE, and some cases of malignant transformation of OLL and OLP may occur via clinical transformation to OE. As chronic inflammation is implicated in the etiology of oral cancer, it may be a local factor that modulates the progression of OPMDs such as OLD and OE [29]. Of note, the erythematous/atrophic clinical presentation that is commonly found in OLD, may sometimes cause diagnostic difficulties clinically, but should not be confused with OE.

Interestingly, it was reported that local irritation from dentures produced a reversible lesion clinically identical to erythroplakia (sharply demarcated fiery red area situated at 0.1–0.2 mm lower level compared to the surrounding mucosa) but with also lichenoid features in the adjacent mucosa in two patients [6]. In some of our cases, minor white areas at the periphery of the OE and OELSCC lesions were present, and this feature could be seen both in the cases where the patient had an OLD, and where the patient did not have another oral mucosal disease. Of note, local irritation was not detected in the present cases and the lesions were persistent.

About 45% of the present OEs were located in the buccal mucosa which is among the most frequent sites of OE [24]. Higher proportion of the present OEs were located in the gingiva (27%) and tongue (18%) than previously reported [7] although ventral tongue is mentioned as a predilection site of OE in the WHO Histological typing of cancer and precancer of the oral mucosa [30]. Floor of the mouth (FOM) is considered one of the most common locations for OE [7] but none of the present cases was seen in this site. Although tongue is a predilection site for oral SCC, gingiva was the most common location of OELSCCs in the present study. Floor of the mouth is one of the most common locations for oral SCC, but only one of the OELSCCs was located in the FOM (in a smoker). As smoking and alcohol use are considered risk factors especially for FOM oral cancers, the relatively small proportion of patients having these habits could partly explain this discrepancy in the present case series. However, the small number of cases prevents any reliable conclusions about the matter.

Most of the present OEs were larger than the earlier reported typical diameter of < 1.5 cm [7]. The size of OELSCCs was comparable to the OEs, although none of the OELSCCs were less than 15 mm in diameter.

The surface of OEs may be smooth or granular [8]; all our OE cases had a smooth surface. Some of the OELSCCs had an uneven or granular surface (Fig. 2). The vast majority of the present OEs had well-defined borders all around, which is a recognized feature of OEs [8]. Also most (but a smaller proportion than of OEs) of the OELSCCs were well-defined. One of the OELSCCs was poorly-defined. A sharp demarcation from the surrounding mucosa is considered an important clinical differential diagnostic feature of OE [6], as erythema that is associated with reactive or inflammatory lesions of the oral mucosa has almost always diffuse borders (common exception to this is geographic tongue). OEs may be flat or situated at a slightly lower level than the adjacent mucosa [4, 6, 28] and both presentations were seen in the present OE and OELSCC cases (one OELSCC case was considerably depressed below the adjacent mucosa). OEs are soft on palpation and induration indicates the development of invasive carcinoma [7].

Often the occurrence of symptoms in OE is not reported in studies, so the exact prevalence of these in OE is difficult to estimate. In our series, less than a third of patients had symptoms associated with OE. In contrast to this, the vast majority of OELSCC patients experienced symptoms. Symptoms that have been reported in association with OE include irritation, pain, burning, dysphagia and slight itching [28, 31–33].

Over 80% of the OEs presented histopathologically with dysplasia and over 60% were diagnosed as severe dysplasia or carcinoma in situ. This finding is in line with the literature [11]. In their series of 8 OEs, de Azevedo et al. found that 62.5% of OEs were histopathologically severe dysplasia or carcinoma in situ, 25% were moderate dysplasia and 12.5.% showed no dysplasia [34]. On the other hand, in a series of 15 OEs (of which all were biopsied but only 9 surgically treated), 26% were histopathologically severe dysplasia or CIS, 40% were moderate dysplasia, 33% were slight dysplasia and 1% were non-dysplastic [35]. It is still a matter of controversy whether oral epithelial carcinoma in situ represents a precancerous or a cancerous lesion. In the present study, we classified the lesions with carcinoma in situ as erythroplakias according to the WHO Classification of Head and Neck Tumours (2017) where carcinoma in situ in the oral cavity is defined as synonymous to severe dysplasia [24].

It is noteworthy that the histopathologic diagnosis of first diagnostic biopsy altered to a more severe histopathologic diagnosis after examining the excision biopsy in a third of the OE cases. This is a finding observed in several previous studies on OPMD [27, 36, 37]. For example, a study where the histopathologic findings of incision and excision biopsies of premalignant lesions were compared found that only 49% of the diagnoses concurred, with 35% changing to a more severe diagnosis [36]. Also two of the present OELSCCs were initially diagnosed as dysplastic/carcinoma in situ, but after excision of the lesion, the diagnosis was invasive carcinoma. It is therefore important to excise every oral lesion diagnosed as erythroplakia irrespective of the incisional biopsy diagnosis if the lesion does not resolve after elimination of possible irritants.

The use of adjunctive diagnostic tests, such as vital staining or light-based detection to aid in biopsy site selection may be considered by expert clinicians, especially in large lesions suspected to be OE or OELSCC. It should be noted that there is no evidence for the usefulness of these diagnostic aids in the primary care setting and that they should not be used as a replacement for biopsy [38, 39].

Spontaneous resolution of OE has been observed in a longitudinal study [35] but the natural evolution of OEs is unknown and cannot be reliably predicted in individual cases. Although the evidence base for medical or surgical intervention in preventing malignant transformation of OE is low or non-existing [40], excision is often recommended for at least OEs containing moderate to severe dysplasia/carcinoma in situ [1, 7, 41]. Of note, excision of the whole OE lesion when possible would be justifiable for diagnostic purpose. Also modification of known life-style risk factors is recommended for OE patients [40]. All the present OEs were treated with either surgical excision or laser evaporation. One of the OEs with no dysplasia was followed for 52 months but remained clinically unchanged until it was eventually treated with CO2-laser with no recurrence.

Recurrence of OE was observed in close to half of the present cases. Previous studies have shown also relatively high recurrence rates of 17–53% after excision of OE [27, 37, 42]. In one study, the large size of OE (over 80mm^2^) was the only independent factor that predicted postoperative recurrence [27]. A long-term follow-up of OE is indicated even after successful complete excision [36].

Although OE is thought to have a considerably high MTR of 33–45% [3], a recent meta-analysis estimated a much lower MTR of 19.9% [34], stating that to assess reliably the malignant development of OE in studies, the initial biopsy should rule out the presence of SCC and a clinical follow-up period is necessary. In the present series, malignant transformation occurred in one patient histopathologically diagnosed with severe dysplasia, giving a MTR of 9% in a mean follow-up period of 73 months. The small size of the present study may explain the low MTR observed.

Among the limitations of this retrospective study is that information about some patient characteristics such as alcohol use or symptoms was not available of all cases and that the follow-up period was short in some cases. Due to rarity of OE and OELSCC, the number of cases in this study is limited and therefore it is not possible to make definitive conclusions about the possible clinical differences between OE and OELSCC.

## Conclusions

The definition of OE is still unsatisfactory. Clinically OE-like lesion that has invasive SCC on first incision biopsy/biopsies or excision biopsy, should not be called OE nor classified as an OPMD, nor included in studies as such. The definition and nomenclature of mixed red and white OPMD lesions as erythroleukoplakias or leukoerythroplakias depending on the predominant appearance could possibly clarify the classification of OPMDs further.

There are patients with OE or OELSCCs in whom typical predisposing factors tobacco, areca nut and alcohol use are not involved. Previous OLD seems to be associated with OE and OELSCC in a proportion of patients.

OELSCCs may be more often symptomatic and have more often an uneven surface or ulceration than OEs. These features could help clinicians in assessing the risk of SCC when first encountering a patient with an erythroplakia like lesion in the oral mucosa. Biopsy/biopsies are required for the diagnosis of OE and only the excision of OE may enable the correct diagnosis to be made.

In the future, larger well-characterized patient populations with OEs are needed to elucidate the etiological factors, natural history, best treatment options and prognosis of this rare oral potentially malignant lesion.

### Electronic supplementary material

Below is the link to the electronic supplementary material.

Supplementary Material 1

## Data Availability

The datasets used and/or analysed during the current study are available from the corresponding author on reasonable request.

## Abbreviations

FOM: Floor of mouth
MTR: Malignant transformation rate
OE: Oral erytroplakia
OELSCC: Clinically oral erytroplakia like squamous cell carcinoma
OL: Oral leukoplakia
OLD: Oral lichenoid disease
OLL: Oral lichenoid lesion
OLP: Oral lichen planus
OPMD: Oral potentially malignant disorder
SCC: Squamous cell carcinoma
WHO: World Health Organization
