# Supplementary material for: Oral erythroplakia and oral erythroplakia-like oral squamous cell carcinoma – what’s the difference?
Source: BMC Oral Health. 2023 Nov 13;23:859. doi: 10.1186/s12903-023-03619-2 (PMC10644603; doi:10.1186/s12903-023-03619-2)
Supplement: Supplementary file 1 — Supplementary Material 1 [file 12903_2023_3619_MOESM1_ESM.docx]

Supplementary Table. Definition/classification and characterization of oral erythroplakia in pertinent literature.

| Author | Definition | Additional clinical description | Further description relating to OE | Comments |
| --- | --- | --- | --- | --- |
| Shear (1972) | “Classification of oral erythroplakia is proposed based on the clinical variations: 1) Homogeneous erythroplakia 2) Erythroplakia interspersed with patches of leukoplakia 3) Granular or speckled erythroplakia (embracing the lesion described as speckled leukoplakia)” | - | “Microscopic variations of *erythroplakic* lesions are presented as: 1) Neoplastic (Squamous carcinoma, carcinoma-*in-situ* (intra-epithelial carcinoma) and less severe forms of epithelial atypia) 2) Inflammatory (Candida albicans infections (including denture stomatitis), tuberculosis, histoplasmosis, miscellaneous specific, non-specific and non-diagnosable lesions)” | - |
| Kramer et al. (1978) | “The term erythroplakia is used analogously to leukoplakia to designate lesions of the oral mucosa that present as bright red, velvety plaques which cannot be characterized clinically or pathologically as being due to any other condition” | “Occasionally, the surface is nodular and there may be white or yellow spots on the red background. In other cases the red areas of erythroplakia may be intermingled with white patches of leukoplakia.” | “Just as there are many oral lesions that present clinically as white patches on the mucosa, so there are a number of conditions that appear as red areas. These include some dermatoses, inflammatory conditions due to local infection, or a more general subacute or chronic stomatitis associated with the presence of dentures, tuberculosis, fungal infections, and other conditions. Some red plaques prove to be early squamous-cell carcinomas. The red patches that cannot be classified in any of these categories fall into the erythroplakia group.” | Figure shows a homogenously red, mostly well-defined lesion in the buccal mucosa. |
| Axéll et al. (1984) | “The term erythroplakia is used analogously to leukoplakia to designate lesions of the oral mucosa that present as bright red patches or plaques that cannot be characterized clinically or pathologically as any other condition” | “A complete description of a precancerous lesion (e,g, leukoplakia and erythroplakia) comprises aetiological, clinical, topographical, and histological characteristics.”  “These characteristics were discussed only as related to leukoplakia.” | 2. Clinical description  “The clinical subdivision of precancerous lesions has important implications for prognosis. It is generally agreed that the homogeneous leukoplakia carries the lowest risk of malignant transformation, whilst non-homogeneous forms, mixed red- and whitish lesions and pure erythroplakias carry a higher risk.  a) Homogeneous leukoplakia (simplex) A uniformly whitish lesion with a smooth or corrugated surface,  b) Non-homogeneous leukoplakia.  i) Erythroleukoplakia (erosive leukoplakia). A whitish lesion that includes red areas.  ii) Nodular leukoplakia. A lesion with slightly raised, rounded, red and/or whitish excrescences that may be described as granules or nodules.  iii) Verrucous leukoplakia. An exophytic lesion with irregular sharp or blunt projections.  NB. Erythroleukoplakia and the nodular type of leukoplakia are also referred to as speckled leukoplakia or as speckled erythroplakia. Erythroleukoplakia and the nodular leukoplakia are in most instances associated with candidal infection.” | - |
| Axéll et al. (1996) | “The term erythroplakia is used analogously to leukoplakia to designate lesions of the oral mucosa that present as red areas and cannot be diagnosed as any other definable lesion” | - | “Oral leukoplakia is a predominantly white lesion of the oral mucosa that cannot be characterized as any other definable lesion; some oral leukoplakias will transform into cancer.  Clinical variants of white and red lesions of the oral mucosa:  (1) Homogeneous leukoplakia:  A predominantly white lesion of uniform flat, thin appearance that may exhibit shallow cracks and has a smooth, wrinkled or corrugated surface with a consistent texture throughout.  (2) Non-homogeneous leukoplakia:  A predominantly white or white and red lesion (erythroleukoplakia) that may be irregularly flat, nodular or exophytic. The nodular lesions have slightly raised, rounded, red and/or white excrescences and the exophytic lesions have irregular blunt or sharp projections.” | - |
| Pindborg et al. (1997) | “A fiery red patch that cannot be characterized clinically or pathologically as any other definable lesion” | “Some erythroplakias are smooth, and some are granular or nodular. Often there is a well-defined margin adjacent to mucosa of normal appearance.” | - | Figure shows a well-defined red lesion in the floor of the mouth. |
| Barnes et al. (2005) | “The principal oral and oropharyngeal lesions which may be precursor lesions are white patches (leukoplakia) and red patches (erytroplasia/erytroplakia) or mixed red and white lesions” | - | “Red and mixed lesions (speckled leukoplakia) show a higher frequency of dysplasia, often of higher grade” | Figure legend: “Erytroplasia/erytroplakia associated with oral lichen planus (precancer).” |
| Warnakulasuriya et al. (2007) | “The 1978 WHO definition is still current and widely used: ‘A fiery red patch that cannot be characterized clinically or pathologically as any other definable disease’” | “Erythroplakia is often flat with a smooth or granular surface” | “Erythroplakias seem to be relatively uncommon on their own and often present as mixed red-and-white lesions. These should be considered under the term ‘erythroleukoplakia.’’ | - |
| Diz et al. EAOM (2011) | “Erythroplakia has been defined as ‘a fiery red patch that cannot be characterized clinically or pathologically as any other definable disease’” | “The clinical appearance of erythroplakia (fiery red) may be flat or even depressed with a smooth or granular surface (Fig. 5).”  “Generally solitary presentation.” | “Non-homogeneous varieties (of leukoplakia) include: speckled: mixed, white and red (‘erythroleukoplakia’), but retaining predominantly white character.  In the case of a mixture of mostly red and some white changes such lesion is usually categorized as non-homogeneous leukoplakia (‘erythroleukoplakia’).” | Figure shows well-defined lesion in the palatal mucosa, with a possibly ulcerated, fibrin covered area. |
| Scully (2013) | “Erythroplakia: defined by the World Health Organization (WHO) as ‘any lesion of the oral mucosa that presents as bright red velvety plaques which cannot be characterized clinically or pathologically as any other recognizable condition’” | “Flat red plaque”  ”*Erythroplastic* lesions are well-defined velvety red plaques….”  “Red velvety patch of variable configurations, usually level with or depressed below surrounding mucosa.“ | “Some erythroplakias are associated with white patches, and are then termed speckled leukoplakia or erythroleukoplakia.” | Figure shows a bright red lesion in the buccal mucosa.  In 2^nd^ ed. of the book (2008) figure shows red lesion with lichenoid striae around the periphery of the lesion. Figure legend states: “Carcinoma developed in this patient, who actually had long-standing lichen planus with lichenoid dysplasia.” |
| Neville et al. (2016) | “A red patch or plaque that cannot be clinically or pathologically diagnosed as any other condition” | “A well-demarcated erythematous patch or plaque with a soft, velvety texture“ | “May be associated with an adjacent leukoplakia (erytroleukoplakia)” | Two figures that show erythematous macule/patch (one in the floor of the mouth and one in the palatal mucosa) that seem partly well- and partly diffusely defined. |
| El-Naggar et al. (2017) | “’Leukoplakia’ is a clinical term used to describe white plaques of questionable risk once other specific conditions and other OPMDs have been ruled out. Leukoplakias can be homogenously white or predominantly white with nodular, verrucous or red areas. Predominantly white examples with red areas are called erythroleukoplakias (speckled leukoplakias). Oral erythroplakia is defined equivalently, but as a red patch.” | - | - | Defined in relation to leukoplakia, and this implies that oral erythroplakia is defined as a homogenously red or a predominantly red lesion. |
| Regezi et al. (2017) | “Erythroplakia refers to a red patch on oral mucous membranes” | “A velvety red patch with well-defined margins.”  “Focal white areas representing keratosis may be seen in some lesions (erythroleukoplakia).” | - | Two figures of oral erythroplakia that present with focal white areas. |
| Odell (2017) | “A predominantly red lesion of the oral mucosa that cannot be characterized clinically or pathologically as any other definable lesion” | “The term *erythroplasia* is sometimes used to indicate that these lesions are often not raised plaques like leukoplakias, but flat or slightly depressed. Pure red lesions are rare…”  “The surface is frequently velvety in texture and ranges from dull matte red to bright scarlet. The margin may or may not be sharply defined.” | - | Figure shows well-defined red lesion in tongue. |
| Warnakulasuriya (2018) | “The term *erythroplakia* is used analogously to *leukoplakia* and has been deﬁned as “a ﬁery red patch that cannot be characterized clinically or pathologically as any other deﬁnable disease”” | “The lesions of erythroplakia are usually  irregular in outline, although well defined, and have a bright red velvety surface. Occasionally, the surface is granular.” | “Homogeneous leukoplakias are uniformly flat and thin, have a smooth surface, and may exhibit shallow cracks. Nonhomogeneous varieties comprise 3 clinical types and are usually symptomatic:  1. Speckled—mixed, white and red in color (also termed *erythroleukoplakia*), but retaining predominantly white coloration…”  “Mixed white-and-red lesions, previously referred to  as *speckled leukoplakia*, are now termed *erythro-*  *leukoplakia.*”  “Erythroleukoplakia, unlike leukoplakia or erythroplakia, may have an irregular margin.” | Figure shows partly well-defined lesion in palatal mucosa with white areas around the periphery of the red patch. |
| Holmstrup (2018) | “It is widely accepted that the term erythroplakia should be used to recognize fiery red patches that cannot be characterized clinically or pathologically as any other definable lesion (Kramer et al., 1978; Reichart & Philipsen, 2005).” | “Could it be that sharply demarcated, smooth, velvety, or granular fiery red lesions, which are situated slightly below the level of the surrounding mucosa, should be denoted erythroplakias independent of the simultaneous presence of other oral mucosal disorders?” | “Could it be that a supplementary finding of some degree of epithelial dysplasia should be a diagnostic criterion of oral erythroplakia to limit the diagnosis to lesions with a malignant potential?” | A previous report by the author described *erythroplakic* lesions in patients with oral lichen planus (Holmstrup & Pindborg, 1979). |
| van der Waal (2018) | “A red lesion of the oral mucosa; other, well-deﬁned red lesions have been excluded clinically, histopathologically or by the use of other diagnostic aids” | - | “*Leukoplakia:* A predominantly white lesion of the oral mucosa that cannot be wiped; other, well-defined predominantly white lesions have been excluded clinically, histopathologically, or by the use of other diagnostic aids.”  “Clinical classification of leukoplakia  *Homogeneous:* Homogeneous white color; the thickness and texture may vary from thin, smooth, wrinkled, and corrugated to thick and/or verrucous  *Nonhomogeneous:* Mixed white-and-red appearance (“erythroleukoplakia”); the surface may vary from smooth to speckled, granular, or nodular.” | - |
| Woo (2019) | N/a | “A red pebbly, granular plaque” | - | Figure legend states: “Erythroplakia: a mostly red, ulcerated plaque with a small white area on the superior aspect” |
| Warnakulasuriya (2020) | “’A fiery red patch that cannot be characterized clinically or pathologically as any other definable disease’” | “A localized red patch with well defined margins“ | “The term erythroleukoplakia is preferred for mixed white and red patches.”  “Erythroleukoplakia: Mixed, white and red (speckled) but retaining predominantly white character. Margins may be irregular.” | ~~-~~ |
| Warnakulasuriya et al. (2021) | “A predominantly fiery red patch that cannot be characterized clinically or pathologically as any other definable disease” | “A localized red patch with well-defined margins and a matt surface.”  “A solitary lesion.”  “Erythroplakia exhibits a clinical appearance of a sharply demarcated, flat, or depressed, erythematous area of mucosa with a matt appearance.” | “Erythroleukoplakia: Mixed, white and red (speckled) but retaining predominantly white character. Margins may be irregular” | - |

**References:**

**Shear** **M.** Erythroplakia of the mouth. Int Dent J. **1972** Dec;22(4):460-73.

**Kramer** **IR**, Lucas RB, Pindborg JJ, Sobin LH. WHO Collaborating Centre for Oral Precancerous Lesions. Definition of leukoplakia and related lesions: an aid to studies on oral precancer. Oral Surg Oral Med Oral Pathol. **1978**;46:518–39.

**Axéll T,** Holmstrup P, Kramer IRH, Pindborg JJ, Shear M. International seminar on oral leukoplakia and associated lesions related to tobacco habits. Community Dent Oral Epidemiol. **1984;**12:145–154.

**Axéll T**, Pindborg JJ, Smith CJ, van der Waal I. Oral white lesions with special reference to precancerous and tobacco- related lesions: conclusions of an international symposium held in Uppsala, Sweden, May 18-21 1994. International Collaborative Group on Oral White Lesions. J Oral Pathol Med. **1996**;25:49–54.

**Pindborg JJ,** Reichart PA, Smith CJ, van der Waal I. **(1997).** World Health Organization International Histological Classification of Tumours. Histological Typing of Cancer and Precancer of the Oral Mucosa. Berlin, Heidelberg, New York: Springer-Verlag.

**Barnes L**, Eveson J, Reichart P & Sidransky D. **(2005)**. World Health Organization classification of tumours: pathology and genetics of head and neck tumours, 3th ed. IARC Press, Lyon.

**Warnakulasuriya S**, Johnson NW, van der Waal I. Nomenclature and classification of potentially malignant disorders of the oral mucosa. J Oral Pathol Med. **2007** Nov;36(10):575-80.

**Diz P**, Gorsky M, Johnson NW, Kragelund C, Manfredi M, Odell E, Thongprasom K, Warnakulasuriya S, Bagan JV, van der Waal I. Oral leukoplakia and erythroplakia: a protocol for diagnosis and management. EAOM - diagnostic and therapeutic protocols Oral leukoplakia and erythroplakia.

**Scully C. (2013).** Oral and Maxillofacial Medicine: The basis of diagnosis and treatment. 3rd ed. Churchill Livingstone.

**Neville BW**, Damm DD, Allen CM and Chi AC. **(2016)** Oral and Maxillofacial Pathology. 4th ed. Elsevier, St. Louis.

**El-Naggar AK**, Chan JKC, Grandis JR, Takata T, Grandis J, Slootweg PJ (eds) **(2017).** WHO classification of head and neck tumours. Pathology and genetics of head and neck tumours. 4th ed. IARC Press, Lyon.

**Regezi JA**, Sciubba J, Jordan RCK. **(2017)**. Oral pathology. Clinical-pathologic correlations. 7th ed. Elsevier, St Louis.

**Odell EW. (2017)** Cawson’s Essentials of Oral Pathology and Oral Medicine. 9th ed. Elsevier.

**Warnakulasuriya S.** Clinical features and presentation of oral potentially malignant disorders. Oral Surg Oral Med Oral Pathol Oral Radiol. **2018** Jun;125(6):582-590..

**Holmstrup P.** Oral erythroplakia-What is it? Oral Dis**. 2018** Mar;24(1-2):138-143.

**van der Waal I.** Historical perspective and nomenclature of potentially malignant or potentially premalignant oral epithelial lesions with emphasis on leukoplakia-some suggestions for modifications. Oral Surg Oral Med Oral Pathol Oral Radiol. **2018** Jun;125(6):577-581.

**Woo SB.** Oral Epithelial Dysplasia and Premalignancy. Head Neck Pathol. **2019** Sep;13(3):423-439.

**Warnakulasuriya S.** Oral potentially malignant disorders: A comprehensive review on clinical aspects and management. Oral Oncol**. 2020** Mar;102:104550.

**Warnakulasuriya S**, Kujan O, Aguirre-Urizar JM, Bagan JV, González-Moles MÁ, Kerr AR, Lodi G, Mello FW, Monteiro L, Ogden GR, Sloan P, Johnson NW. Oral potentially malignant disorders: A consensus report from an international seminar on nomenclature and classification, convened by the WHO Collaborating Centre for Oral Cancer. Oral Dis. **2021** Nov;27(8):1862-1880.
